# Breaking the cycle of parasitic diseases with edutainment: The intersection of entertainment and education

**DOI:** 10.1371/journal.pntd.0013072

**Published:** 2025-05-28

**Authors:** Muhammad Furqan Arshad, Ibrahim Abbas, Francesca Porcu, Alessandro Ricci, Gabriella Gaglio, Emanuele Brianti, Silvia Carta, Lia Cavallo, Claudia Tamponi, Simona Gabrielli, Camila González, Cinzia Cantacessi, Antonio Scala, Antonio Varcasia

**Affiliations:** 1 Department of Veterinary Medicine, University of Sassari, Sassari, Italy; 2 Parasitology Department, Faculty of Veterinary Medicine, Mansoura University, Mansoura, Egypt; 3 Department of Medical and Surgical Sciences, University of Cagliari, Monserrato, Italy; 4 Department of Veterinary Sciences, University of Messina, Messina, Italy; 5 Department of Public Health and Infectious Diseases, Sapienza Università di Roma, Roma, Italy; 6 Universidad de Los Andes, Bogota, Colombia; 7 Department of Veterinary Medicine, University of Cambridge, Cambridge, United Kingdom; Mizan-Tepi University, ETHIOPIA

## Abstract

Parasitic diseases represent a substantial public health challenge worldwide. Traditional educational strategies have often fallen short in driving sustained behavioral shifts that are nonetheless essential for reducing the burden of these diseases. Edutainment, a blend of education and entertainment, is the synthesis of pedagogical content with recreational frameworks, leveraging narrative and visual appeal to elevate the learning experience through enriched experiences, aligning with the principles of “warm cognition”. Human cognitive processes, including attention, learning and memory, are influenced by emotions. As a result, emotional experiences are remembered vividly and accurately, with great resilience over time. Several edutainment approaches have been successfully utilized to inspire positive behavioral changes against soil-transmitted helminths (STHs), schistosomiasis, echinococcosis, and other diseases. This scoping review delves into several documented approaches with sustainable positive post-intervention outcomes. Approaches such as animated cartoons, gamification, songs, videos, and music, mobile health applications, hands-on experience, posters, comics and educational booklets, puppet shows, toy animals, cardboard and plastic-coated drawings, drawing activities and competitions, group discussions, illustrated booklets and questionnaires have yielded statistically significant improvements in participant’s knowledge related to parasitic diseases (up to 60% increase in knowledge scores), alongside notable reductions in risks of parasite transmission and infection prevalence. These improvements highlight the potential of edutainment to enhance community awareness, promote long-term behavioral changes, and ultimately contribute to reducing spread of disease. Moreover, artificial intelligence (AI) can be integrated into edutainment approaches to meet the growing demand for personalized and effective learning methods. We argue that such AI-driven edutainment can underpin sustainable progress in the control of parasitic diseases.

## Introduction

Parasitic diseases represent a substantial global public health challenge, especially in regions characterized by inadequate sanitation and limited access to healthcare. Among these, soil-transmitted helminths (STHs) (e.g., hookworms, roundworms, and whipworms) affect more than 1.5 billion people globally [1–3] and waterborne schistosomiases caused by blood-flukes of the genus *Schistosoma* are responsible for over 200,000 deaths annually, predominantly in sub-Saharan Africa [4]. Children are more likely to acquire these infections due to various factors including close contact with pets, weaker immune systems, poor hygiene, and higher exposure to parasite infective stages. For instance, a 48% prevalence of infections by STHs was recorded in 61,690 children examined from 1997 to 2020 in Ethiopia, confirming the extent of the issue [5]. In addition, Cystic Echinococcosis (CE), a neglected disease according to the World Health Organisation (WHO), represents the second most important food-borne parasitic disease and remains a major public health problem, due to its zonal endemicity and potential for significant morbidity. From a One Health perspective, the prevention and control of these diseases are key WHO objectives [6]. This requires the implementation of appropriate health education practices to effectively educate children and communities regarding health, hygiene, and the prevention of infections.

Edutainment, the intersection of entertainment and education, has been developed as an instrument for disseminating information, influencing attitudes and behaviors, and promoting health education [7,8]. The term *edutainment* was coined in 1973 by Robert Heyman, a National Geographic filmmaker [9]. Edutainment is an educational paradigm that integrates pedagogical content with elements of entertainment, designed to captivate audience engagement and enhance cognitive retention [10]. Edutainment encompasses a wide range of methodologies, including animated visual media, live simulations, gamification, and narrative-based instruction [11,12]. These approaches hold the potential to target audiences in different educational settings, including educational institutions and community health campaigns, thus addressing various aspects of the prevention of parasitic diseases.

Studies in the areas of neuroscience and psychology have highlighted the interdependence between emotional and cognitive processes, a re-evaluated human dimension in learning. This interdependence leads to analyzing the teaching-learning process from the perspective of *warm cognition* [13], a cognitive state that is influenced by emotions, personal experiences, and social contexts. *Cold cognition*, on the other hand, involve mental processes which are more rational and detached. Children primarily exhibit warm cognition, whereas cold cognition develops with age. Humans not only memorize information, but also emotions [14], that can influence decisions, future actions, and the activation of executive functions in both young and older individuals [13,15].

Children represent ideal targets for the implementation of edutainment strategies, due to their developmental plasticity, long-term multiplier impact, and higher risk profile [16–18]. Children can subsequently be entrusted with the role of health messengers, acting as agents for change, spurring the modifications of health behavior of their families and the wider community [19]. To ensure that children effectively absorb and relay health information, edutainment relies on the cognitive theory of multimedia learning, which optimizes learning by utilizing both visual and auditory channels [20]. This dual-channel strategy has been shown to enhance engagement and memory retention by engaging multiple senses, particularly in age groups where standard education methods may be perceived as ineffective or overly rigid [21].

**Fig 1:**
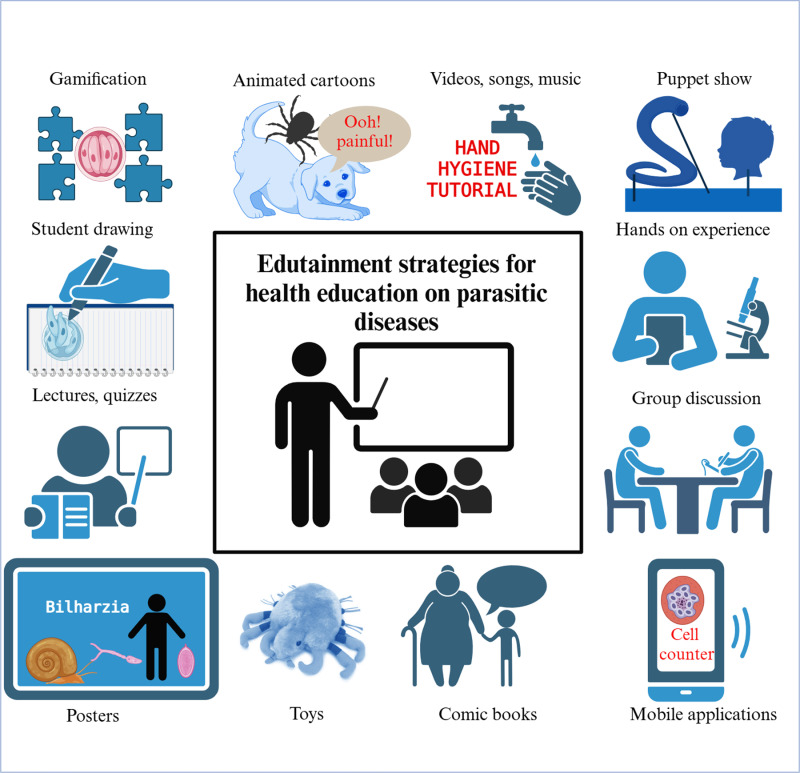
Edutainment strategies for health education on parasitic diseases. (Created with Biorender.com).

The efficacy of edutainment approaches designed to address parasitic diseases is frequently evaluated *via* comparative pre- and post-intervention assessments. Such assessments have demonstrated that these interventions not only enhance participants’ awareness and comprehension but also contribute to long-term behavioral shifts that mitigate disease transmission. In addition to reviewing these pre- and post-intervention assessments, this article provides a comprehensive overview of current edutainment approaches focused on parasitic diseases (Fig 1). This article also discusses the challenges associated with implementation of edutainment approaches, including resource limitations, low literacy rates, and varying cultural contexts, and highlights the need for further innovation, e.g., *via* AI-driven personalization and adaptive learning technologies.

## Methods of the review

### Search strategy

This scoping review was conducted in line with PRISMA guidelines [22], and the protocol was submitted to the Open Science Framework Platform (https://doi.org/10.17605/OSF.IO/HVNYA). A systematic electronic search was conducted twice within 6 months (May–October 2024) to identify and collect all published papers on current edutainment approaches for controlling various parasitic diseases, without restrictions on publication date. Databases consulted included PubMed, Science Direct, and Scopus. Additionally, the database Google Scholar was also consulted to collect gray literature. Search keywords included ‘health education’, ‘edutainment’, ‘entertainment education’, ‘participatory methods’, ‘school-age children’, ‘prevention’, ‘parasites’, ‘parasitic zoonosis’, ‘soil-transmitted helminths’, ‘schistosomiasis’, ‘Cystic Echinococcosis’, ‘animated cartoons’, ‘comic books’, ‘games’, ‘videos’, ‘posters’, ‘illustrations’, ‘puppet shows’, and ‘mobile health applications’. A full list of keywords is provided in S1 Table. Keywords were inserted in the search string in various combinations using the Boolean operators “AND” and “OR” (S1 Table).

### Screening for eligibility and data extraction

Initial title/abstract screening was conducted by two authors (MFA and IA) to identify relevant articles. To assess their eligibility for inclusion in the present review, articles that passed the first screening were subjected to a second round of screening of their full texts by three authors (MFA, IA, and AR). Inclusion criteria were: studies that used any edutainment approach to increase awareness of parasitic diseases in school-age children or other groups, articles available in full text, research articles, and articles published in English. Exclusion criteria were: articles describing edutainment approaches for controlling non-parasitic diseases (e.g., viruses, bacteria, and nutritional deficiencies), articles available in abstracts only, and non-original contributions (e.g., review articles and books). Results obtained by each author tasked with screening were compared, and any discrepancies were resolved by discussion and consensus or by consultation with the senior author (AV). Cohen’s Kappa coefficient (*k*), calculated in Excel, was employed to estimate the degree of agreement between screeners. The values obtained were interpreted as follows: no agreement (*k* < 0.0), slight (0.1–0.20), fair (0.21–0.40), moderate (0.41–0.60), substantial (0.61–0.80), and almost perfect (*k* > 0.80). Data from all eligible studies were extracted by three authors (MFA, IA, and AR) and discussed with CT, CC, and AV. The following data were extracted where possible: study design, target parasite(s), country of origin, audience (e.g., school-age children), edutainment approach used, methods of pre- and post-intervention testing. No statistical methods were used in the present scoping review.

## Results and discussion

A total of 1047 articles were retrieved after our initial database search. Of these articles, 30 were eligible for inclusion in the present review. The remaining articles were excluded (see S1 Fig). Almost perfect agreement was observed between two independent screeners (*k* = 0.85 and 0.87 for first and second screening, respectively) during selection of eligible articles. Overall, these eligible studies originated from Africa (*n* = 12), Asia (*n* = 10), Europe (*n* = 3), and the Americas (*n* = 5), and used various edutainment approaches to increase awareness mostly against STHs. A significant number of studies targeted schistosomiasis and *Taenia solium* cysticercosis. Data from eligible studies are summarized in S2 Table.

### Current study designs for implementing edutainment

Randomized intervention-type studies (controlled or uncontrolled) are often employed to assess the effectiveness of various edutainment approaches in controlling parasitic diseases, particularly STH and schistosomiasis [23]. However, previous controlled intervention trials compared outcomes between two groups only, thus introducing potential selection biases, that are minimized when several groups are compared. Therefore, cluster-randomized intervention-controlled trials are designed to compare the effectiveness of edutainment among several intervention and control groups [24–26]. Most of these randomized trials target school-age children in schools, although community-based studies can also be conducted [27]. Any disparity in age distribution between intervention and control groups represent another potential source of bias [26].

### Edutainment for children

**Fig 2:**
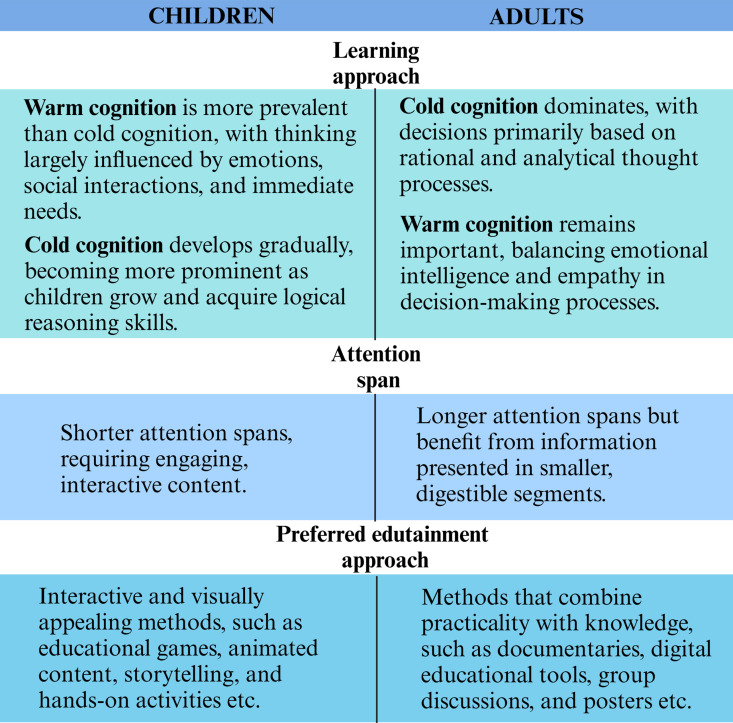
Key differences in learning characteristics of children and adults. (Created with Biorender.com).

Edutainment is defined as the process of purposefully designing and implementing a media message to entertain and educate, in order to increase audience knowledge of educational issue(s), create favorable attitudes, shift social norms, and encourage behavioral change [28]. There is a fundamental difference between teaching children (= pedagogy) and teaching adults (= andragogy) [29]. Key learning differences between children and adults are highlighted in Fig 2. Nowadays, children grow up as digital natives, and thus may highly benefit from edutainment approaches as they coincide with their developmental behaviors, that includes shorter attention spans and a natural preference for engaging, interactive learning experiences [30]. Edutainment engages emotions as well as the intellect [31]. Edutainment aims to ‘be there’, to ‘participate in the experience of knowledge’ and ‘be oneself inside the situation’, while encouraging playing and active involvement [9].

Compared to traditional education methods, edutainment connects fun with learning, making even complex ideas more attractive and digestible [32]. The emotional component of communication plays an important role in learning, both in children and in adults. Human cognitive processes, including attention, learning, and memory, are influenced by emotions [14]. Emotional experiences are remembered vividly and accurately, with great resilience over time. This phenomenon occurs because the brain stimulates an emotional mechanism that activates the learning process. By integrating multimedia, games, and storytelling, learning can be aligned with the mechanisms by which children process and absorb new information, thus resulting in improved retention and comprehension [33]. Furthermore, flexibility is key to ensure wider participation of students with varying attention spans. Active engagement boosts creativity, problem-solving abilities, and social interaction, all pivotal components of a holistic learning experience [34].

### Environment for implementing the edutainment approach

The edutainment approach can be effectively implemented in various environments, including schools, community gatherings, science festivals, and broader public settings [35,36]. In schools, students engage with interactive tools like storytelling, games, and role-playing activities, that make lessons on hygiene, sanitation, and disease prevention more enjoyable [37]. Digital resources like animated videos and mobile applications can visually demonstrate how diseases spread. Activities like puppet shows and competitions can reinforce the lessons on the importance of, e.g., handwashing, safe interaction with animals, and wearing footwear [27].

In community gatherings, edutainment can include street performances and interactive open workshops covering prevention measures in culturally relevant ways. Local radio and television programs can facilitate the dissemination of information, particularly in rural areas, and make information accessible in local languages [28,38]. In workplaces and during public health campaigns, the use of infographics, safety videos, and live demonstrations on the prevention of parasitic and zoonotic disease, sanitation, and hygiene, are a powerful tool to spread awareness [39].

The effective implementation of edutainment strictly depends on well-defined strategies, dedicated facilitators, and the availability of adequate resources. In schools, teachers, health educators, and NGOs play a key role in coordinating edutainment initiatives, utilizing printed materials, digital tools, and interactive teaching methods [40]. Successful implementation may include competitions and quizzes to assess knowledge retention and behavioral change. In community gatherings, healthcare professionals, volunteers, and NGOs drive edutainment efforts, utilizing mobile outreach units and appropriate edutainment strategies tailored to specific contexts [41]. Wherever implemented, continuous assessment of knowledge retention is essential to ensure long-term impact of edutainment approaches.

### Edutainment strategies to increase knowledge of teachers

By identifying the appropriate health needs of a community, priorities for community health programmes can be established, resources can be identified, and community participants can become involved [42]. This process may involve schools and teachers, including questionnaires, to achieve a better understanding of the specific health needs of a given community. Teachers play a key role in health education for their students, and based on their practical experience, they can contribute significantly to the design and development of appropriate educational materials. Collaboration between teachers and researchers ensures that these materials (e.g., illustrated storybooks, animated videos, interactive games, and educational posters) are both pedagogically effective and scientifically accurate, enhancing their impact on student learning and health awareness [43]. Teachers also exert a fundamental role in follow-up strategies, by regularly updating student knowledge of hygiene- and health-related practices [44].

Enhancing teacher knowledge of the target parasite(s) is crucial to the success of the edutainment strategy. For this purpose, several approaches can be implemented; these include the teacher guide, a simple illustrative booklet that includes information on the biology and epidemiology of the target parasite, including routes of zoonotic transmission, as well as risk factors for infection and available control strategies [23,25,35]. The booklets should summarize relevant information on the target parasite(s) using lay language accessible by the general public. In addition, seminars and workshops have been shown to effectively enhance teacher knowledge and encourage their engagement with edutainment programmes [23–25,45]. Similar to the teacher guide, seminars should cover the basic knowledge of the target parasite including the transmission patterns and its prevention [23].

Incorporating new elements into an existing teacher guide can be a complex task, influenced by various factors depending on specific contexts. These challenges often stem from administrative and bureaucratic hurdles, such as obtaining approvals or adhering to institutional protocols. Additionally, the rigid time standards and pre-established schedules within educational systems can further complicate the process, leaving limited flexibility for integrating new content. Moreover, additional classes for health education may represent a burden for teachers and interfere with other forms of schooling. Nevertheless, these classes may be integrated into the school curriculum and regular activities. School health nurses or responsible officers for school health may also participate in health education [46].

### Pre-intervention testing

Pre-intervention testing aims to identify risk factors for parasitic infections, and underpins the selection of the key messages provided to the participants through the education materials [44]. To this end, several approaches have been applied, including Knowledge, Attitude, and Practices (KAP) questionnaires that evaluate participants in relation to the target parasite [35,45]. These structured questionnaires are characterized by a “True False Don’t Know” (TFD) design, with questions posed in layman terms and participants having little to no prior knowledge of the target parasite. Indeed, prior knowledge of the target parasite amongst participants may affect the overall effectiveness of the interventions, and thus it may represent a potential limitation. Questions should cover basic knowledge of the parasite (types of parasitic worms, routes of transmission, symptoms, treatment, and prevention) and hygiene practices (e.g., latrine use, washing hands after defecation and prior to eating, hand-to-mouth contamination, handling food, washing food and/or cooking it thoroughly and wearing footwear). Questions covering sources of information and preferred entertainment media (e.g., favorite television show and/or most appreciated cartoon) can also be included as these may assist the selection of appropriate intervention tools [27]. The pre-intervention questionnaire is developed and piloted in collaboration with other researchers and educators, based on experiences gained over the course of previous edutainment trials [24]. In several previous studies, cross-sectional investigations were conducted to screen the participants to detect prevalence and intensity of the target parasites (e.g., STHs) [23,43], followed by mass anthelmintic (e.g., albendazole or mebendazole) treatment of the participating cohort to exclude potential biases [23,25,45]. Additional testing of soil samples in the participant environment is occasionally conducted. Data collected from such screenings may lead to adaptation of pre-questionnaires to test knowledge of participants regarding specific STH infections and their routes of transmission [i.e., oro-fecal (e.g., whipworms and ascarids) *versus* skin-penetration of infective stages (e.g., hookworms and schistosomes)]. In addition, other means of pre-intervention testing include group discussions [26,44], and drawing activities, in which the participants are asked to draw an illustration of the target parasite, followed by a brief discussion [24,32,47].

Introduction and applications of various edutainment approaches to address parasitic diseases are summarized in Table 1.

**Table 1:** Description, examples, and applications of various edutainment approaches.

Approach	Description	Examples and applications		Link
**Animated cartoons**	Using captivating visuals of parasites and engaging storytelling of different zoonotic infections, animated cartoons simplify complex concepts for children, ensuring better educational retention.	Fight the parasite (Sardinia, Italy)	A project to raise awareness on CE^a^ in schoolchildren.	[35]
		Koko et les lunettes magiques (Côte d’Ivoire)	Conveyed information on STH^a^ infections and associated hygiene practices in the local language.	[44]
		The magic glasses (Languna, the Philippines and China)	To raise awareness about STH^a^ infections among school children.	[24] [48]
**Gamification and computer-based educational tools**	Use of engaging multimedia elements to facilitate understanding and retention of information, while fostering creative thinking and problem-solving. The approach aligns well with the children’s preferences.	The Vicious Worm (Various locations)	A computer-based tool that aims to improve knowledge of *Taenia solium.*	[49] [50] [51]
		Route of infection	In this game, the player must drag the parasite to the appropriate route of entry in the body.	[52]
		Kill the parasites!	In this game, the player must match different parasites and the drugs used against them.	[53]
		No more schisto	Board game to teach students about schistosomiasis infection and treatment, highlighting risky behaviors.	[54]
		Schisto and ladders (Nigeria)	Board game to address schistosomiasis infection.	[55] [56]
		Worm free (Indonesia)	A mobile phone game to teach students about helminthiasis.	[57]
		Fight the parasite project (Sardinia, Italy)	Games designed based on basic information on the risks of CE^a^.	[35]
**Songs, videos, and music**	This multimedia approach with repetitive lyrics, appealing melodies, and visual storytelling can reinforce educational content according to the Social Learning Theory of Albert Bandura, 1963, which asserts that by observing others, people develop behavioral rules that guide their future actions [58].	Edutainment and infographics for schistosomiasis (Ndumo area, Kwazulu-Natal, South Africa)	The study utilized a combination edutainment approach containing a song, an animated video, a drama, and a poem significantly increased children’s understanding of schistosomiasis transmission and prevention.	[59]
		Lawa model (Lawa lake region, Thailand)	Songs and videos in schools contributed to a substantial reduction in the prevalence of opisthorchiasis.	[60]
		Maskandi experience (South Africa)	A cultural song was used to spread awareness on malaria.	[61]
**Mobile health applications**	Offer a significant advantage in health education due to their accessibility and versatility. These apps can provide vital health information, educational flashcards for a range of parasites, and monitoring tools to a broad audience.	CAPC^a^ internal parasites ID	Contains veterinary education flashcards displaying eggs of 100 species of internal parasites.	[62]
		Parasite management guide app.	Provides knowledge of a range of parasitic diseases.	[63]
		Parasit Xpert	A diagnostic tool for the identification of a range of parasites, including zoonotic species.	[64]
		Human parasitology flashcards	An interactive app. designed to familiarize learners with various features of human parasites.	[65]
		Diplemicro	A portable microscope for smartphones.	[66]
**Hands on experience**	This participatory approach is useful to engage students and demonstrate that infections can spread among people through contaminated food and unhygienic attitudes.	Worm Hunters project (Columbia)	To raise awareness about whipworm infections, handshake challenge, and the use of ‘Contamination detectives’, helped in demonstrating how infections spread.	[67]
		Fight the parasite (Sardinia, Italy)	A light microscope was used to visualize *Echinococcus* specimens.	[35]
**Posters**	They are traditional, yet engaging, and can be placed in community gathering spots, ensuring wide outreach. Posters can be displayed on the walls of the classroom after teaching [68].	An extended educational package (Vietnam)	Posters were placed at various locations, sharing knowledge of foodborne zoonotic trematodes.	[69]
		An educational intervention (Izmir, Turkey)	Schools used posters and brochures to raise awareness of CE^a^.	[70]
		A comprehensive approach (Seychelles)	Health education program that employed posters, alongside other health interventions, for effective intestinal parasite control.	[46]
**Comics and educational booklets**	Comics storytelling captivates the attention of young readers, teaching them how outbreaks occur and prevention measures. Collaboration between science experts and artists specialized in science communication [71,72], is key to successfully harness this medium in public health strategy [73].	Fight the parasite (Sardinia, Italy)	Comic booklets were utilized to teach school children about the risk factors and hygiene practices to address CE^a^.	[35]
		Juma na Kichocho (Zanzibar, Tanzania)	To teach school children about urogenital schistosomiasis.	[74]
		Worm Hunters project (Columbia)	To raise awareness about whipworm infections, children received a comic strip as part of the project.	[67]
		Health education learning package (Malaysia)	Various strategies were applied, including a comic book to educate children on STH^a^ infections.	[23]
**Puppet shows, toy animals, cardboard, and plastic-coated drawings**	With interactive visual performance, culturally relevant narratives, and characters, puppet shows are a fun method to provide health information according to social cognitive and norming principles [75]. Props can be used to simulate the parasite life cycle.	Rama and the Worm (Java, Indonesia)	This shadow puppet performance significantly improved knowledge and behaviors related to STH^a^ infections in village people.	[27]
		A project (Puna district, Hawai’i)	Puppet shows were utilized to share information about *Angiostrongylus cantonensis* (rat lungworm), teaching them how to control the parasite.	[76]
		Fight the parasite (Sardinia, Italy)	The key points of the CE^a^ life cycle were represented using cardboard or plastic toys.	[35]
**Drawing activities**	This approach engages children with visual and creative expressions and enhances their ability to understand complex life cycles by retaining information on different developmental stages, e.g., eggs, cysts, oocysts, and adult worms.	(Puerto Iguazú, Misiones province, Argentina)	Drawing activities on intestinal parasites (roundworms and others) significantly enhanced the student knowledge.	[47]
		Fight the parasite (Sardinia, Italy)	896 school children were inspired to create drawings related to CE^a^ following edutainment content..	[35]
		(Yucatan, Mexico)	261 drawings by school-aged children were utilized as tools to assess their understanding of triatomine vectors and Chagas disease.	[77]
**Group discussions**	This approach is effective in reinforcing the educational content by promoting peer learning by shared insights and experiences.	Community perceptions (the Philippines)	Group discussions were used to raise awareness and evaluate community knowledge of schistosomiasis.	[78]
		Focus group discussions (Tanzania)	To assess the community knowledge of soil-transmitted helminths.	[79]
		Explorative study (Colombia)	Group discussions provided insights into the perceptions of patients suffering from Chagas disease, cutaneous leishmaniasis, and leprosy.	[80]
**Illustrated booklets/lectures, Quiz/questionnaire**	Attractive illustrations related to parasites and a final quiz make complex information more accessible and memorable.	Fight the parasite (Sardinia, Italy)	Booklets and engaging lessons were used to raise awareness of CE^a^. Pre- and post-intervention questionnaires were also utilized.	[35]

^a^CE, cystic echinococcosis; STH, soil-transmitted helminths; CAPC, companion animal parasite council

Studies detailing edutainment interventions, target parasites or parasitic diseases, and their quantitative outcomes are presented in Table 2.

**Table 2:** Studies with numerical outcomes of intervention effects.

Reference	Edutainment intervention utilized	Target parasite/parasitic disease	Quantitative intervention outcomes
Porcu and colleagues (2022) [35]	Animated cartoons, games, hands on experience, comics, cardboard and plastic-coated drawings, illustrated booklets	Cystic echinococcosis	Participants demonstrated significant knowledge improvement, with the percentage of correct responses increasing from 65% (pre-intervention) to 87.9% (post-intervention).
Mationg and colleagues (2022) [48]	Educational cartoon video, classroom discussion, comics, drawing competitions, and essay-writing	Soil-transmitted helminths	The edutainment intervention led to a 5.3 percentage point increase in knowledge scores, a 1.1 percentage point improvement in behavior, and a 60% reduction in STH^a^ infection odds in schools with baseline prevalence (≤15%).
Uwibambe and colleagues (2024) [50]	Computer-based educational tool	*Taenia solium* cysticercosis	The training significantly improved participants’ knowledge of *Taenia solium* cysticercosis, with the median score increasing from 15/21 (71.4%) to 19/21 (90.5%).
Mindu and colleagues (2020) [59]	Song, animated video, a drama, and a poem	Schistosomiasis	This health education intervention significantly improved knowledge on schistosomiasis among school children. At post-intervention (a knowledge test), the mean score of the whole sample increased to 15.6/35, from a baseline score of 6.5/35.
Sripa and colleagues (2017) [60]	Participatory community-based intervention	Opisthorchiasis	With the implementation of Lawa model, the infection rate in the 12 villages surrounding the lake declined to <10% from an average of 60%.
Nguyen and colleagues (2024) [69]	Educational leaflets, educational posters, lecture	Clonorchiasis caused *by Clonorchis sinensis*	The edutainment intervention improved clonorchiasis knowledge (OR^a^ = 2.80), attitudes (+0.363), and reduced raw fish consumption (OR = 0.15), enhancing disease prevention.
Albonico and colleagues (1996) [46]	Radio phone-in broadcast, video film, leaflet and poster	Intestinal parasitic infections	The overall prevalence of intestinal parasites in schoolchildren decreased from 60.5% to 33.8%.
Al-Delaimy and colleagues (2014) [23]	A teacher’s guidebook, workshop, posters, comic book, music video, puppet show, drawing activities, and an aid kit.	Soil-transmitted helminths	Reinfection rates after 6 months of intervention were significantly lower in the test group compared to the control group: Trichuriasis (79.3% vs. 83.0%), Ascariasis (63.3% vs. 82.3%), and mixed infections (39.6% vs. 75.5%).
Kurscheid and colleagues (2018) [27]	Shadow puppet production	Soil-transmitted helminths	The study showed improvements in both knowledge (from 48.6% to 62.8%) and behavior (from 77.4% to 80.6%) regarding gastrointestinal and helminth diseases post-intervention.

^a^STH, soil-transmitted helminths; OD, odds ratio

### Post-intervention testing

Post-intervention aims to evaluate edutainment outcomes, including knowledge gain, cognitive advancement, and behavioral changes of the participants. Post-intervention testing approaches include TFD questionnaires, that should be delivered as soon as possible following completion of intervention and be identical to pre-intervention assessments [35,44,59]. Both pre- and post-intervention questionnaires are assessed using a scoring system that enumerates correct answers [27], and comparisons between pre- and post-intervention answers should be conducted using appropriate statistical tools. In addition, post-intervention screening of both participants and environment may be required in some instances (e.g., STHs) to evaluate any reduction in prevalence and intensity of the target parasites post-intervention [23–25,45,46].

Furthermore, a sheet containing illustrations depicting hygiene habits to control parasitic diseases can also serve as a post-intervention strategy to evaluate edutainment outcomes. In this approach, participants indicate whether any of the represented activities constitute (or not) appropriate preventative measures by drawing happy or sad faces next to each illustration [47]. Conversely, changes in participant behavior (e.g., washing hands after using the toilet at school) are usually observed by research staff and occasionally by trained students [24]. However, in studies characterized by large sample sizes, any behavioral changes rely on participant self-reporting, that may introduce measurement errors [25].

Surveys can provide valuable insights into perceptions and behaviors [81]. Pre- and post-intervention quizzes, as well as satisfaction questionnaires, represent useful tools to acquire data aimed to evaluate the effectiveness of structured edutainment-style projects in comparison to mainstream models, and to assess the acquisition of KAP competences of students at all levels and the involvement of both the teaching staff and the community. Questions must be structured in collaboration with teams of pedagogues, parasitologists, and biologists and formulated appropriately, in order to ensure the reliability of any information supplied to decision-makers [81].

### Potential limits

By combining educational content with engaging formats, multiple studies have reported significant improvements in knowledge retention and positive behavioral change. Key approaches, including interactive games, animated videos, mobile applications, and educational posters have been successfully applied in various settings. While edutainment offers innovative methods for addressing parasitic diseases, it is essential to acknowledge the potential constraints of each approach.

Despite their effectiveness, the production and distribution of educational cartoons require significant resources and/or creative skills, both of which are not readily available (particularly in low-income countries were parasitoses are often endemic); in addition, the impact of this approach may be limited unless fully integrated with other educational and health initiatives such as deworming programs. On the other hand, potential limitations to the use of gamification and computer-based educational tools include the need to access digital devices and the challenge of maintaining children attention in the long term. Additionally, digital games require frequent updates and validation to remain relevant and accurate.

Songs, videos, and music-based approaches rely substantially on the quality of the content, and ensuring access to high-quality content in resource-limited settings may prove challenging. Moreover, such approaches are considered ‘passive’ and thus should be integrated with other edutainment methods. In the post-Covid era, educationally useful videos in relation to hand hygiene are widely available; however, such videos are most effective when aligned with social media best practice principles to avoid miscommunication [7]. The application of mobile health applications for the dissemination of information is an effective strategy; however, barriers such as access to smartphones and literacy levels in low-income countries may severely limit the reach and effectiveness of health educational initiatives.

Posters attract passive engagement with limited depth of information, and thus may be relatively ineffective in areas of the world characterized by low literacy rates as they rely on visual appeal and literacy. Compared with other interactive or hands on approaches, comic books may not fully address diverse learning preferences. Challenges such as varying cultural contexts and literacy levels may limit their accessibility and reduce their overall impact. Health information professionals play a key role in identifying, cataloguing, indexing and promoting educational health comics, thus contributing to improved knowledge and access to health information in a comics format. It could also contribute to changing perceptions and encouraging both patients and family members, as well as health care workers, to appreciate comics as a credible and potentially valuable form of health information [71].

The application of puppet shows as an edutainment strategy is effective, although potential challenges include the need for continuous content updates to reflect the latest scientific knowledge and for ensuring cultural relevance to different populations. Additionally, on their own, puppet shows may prove insufficient and should be complemented with other educational interventions. Drawing activities and use of cardboard props are effective educational interventions, although they depend on the artistic skills of learners and their ability to accurately depict scientific concepts, that may rely on the availability of skilled facilitators.

Group discussions require skilled facilitators to ensure focused and informative discussions and avoid misinformation. In educational settings with large class sizes, active participation of all children may prove challenging. If booklets/lectures are used, it is important to ensure that they are culturally pertinent and age appropriate, and that the questionnaires are designed to accurately assess comprehension while avoiding frustration or disengagement. The effectiveness of this approach may be constrained by variations in literacy levels among the child population.

### Future directions

Artificial intelligence (AI) has successfully modernized the educational system and contributed to the evolution of society 5.0 by embracing innovative practices [82]. Globally, the use of AI-based automated systems in education is accelerating due to effectiveness and the growing demand for personalized learning. The advantages of integrating such systems with edutainment approaches include various cognitive benefits [83]. The idea of leveraging the benefits of AI to enhance the effectiveness of edutainment approaches is in its infancy, and it is likely that this will lead to a paradigm shift in the field of education by enhancing engagement, personalization, and knowledge retention.

Digital game-based learning represents an effective approach to knowledge acquisition, with various examples already in practice, e.g., ‘Parasite Patrol’ and ‘ZooTrivia’ [84,85]. AI integration in these games is forthcoming, and will allow users to interact with the life cycles and transmission routes of a range of pathogens in realistic environments, thus facilitating a deeper understanding of their effects on humans and animals [86,87]. The personalization capability of machine learning supports content tailored to the specific needs of each learner, adjusting levels of difficulty, and providing feedback on user performance [88]. Moreover, its adaptive learning systems allow to regularly refine contents based on the identifications of knowledge gaps. Conversational agents powered by natural language processing can simulate interactive dialogs, allowing children and community members to engage in real-time discussions on disease prevention. These AI chatbots can answer queries, provide scenario-based learning experiences, and reinforce key messages in an engaging manner [89].

With AI-driven virtual and augmented reality, immersive environments can be created where students can work with high-resolution models of parasites [90]. AI-powered intelligent tutoring systems are being applied in real-time support, thus helping to achieve sustainable education [91]. By integrating AI in edutainment, educators will create interactive and engaging learning environments that will substantially facilitate the acquisition of knowledge and raise student awareness of parasitic and zoonotic infections, thus providing support toward better public health outcomes.

The incorporation of participatory approaches in University curricula will have a long-term impact on the veterinary profession, among others [92]. Universities, as primary channel for academic knowledge dissemination, are amongst the frontline interlocutors for science communication communities *via*, for instance, institutional communication and the dissemination of research projects. Thus, a virtuous circle of social media supporting the dissemination of Science Communication (SC) may be created, with wider public outreach. In addition, academic training should include pathways or educational activities that promote the concept of SC and associated skills [93].

The integration of edutainment tools in the school curriculum and the assessment of student achievement is also crucial. Schools represent formal educational institutions that offer facilities to support or counteract successful health education and health promotion processes. The edutainment-style approach allows to effectively convey key health information, even with limited resource availability and/or in economically and socially challenging settings. Social factors collected by ISTAT (micro-criminality, homicide rate, cultural demand) confirmed the association between zoonosis disease risk (e.g., CE) and critical rural conditions [94]. Targeted One Health interventions are crucial in high-risk areas. Furthermore, interventions must be envisaged as long-term programmes, structured and fully integrated within the school curriculum and subjected to formal assessment. This long and complex process must be (conceptually and financially) supported by relevant government and higher-education institution stakeholders, such as Ministry of Health, Universities and local municipalities.

## Conclusion

Edutainment is a promising approach to address the burden of parasitic diseases, with documented potential to enhance knowledge and encourage positive behavioral changes among target audiences, particularly school children. Engaging educational interventions have been applied to a variety of projects aimed to raise awareness on common parasitic challenges including STHs, schistosomiasis, echinococcosis and others. Post-intervention testing has demonstrated that modern edutainment approaches such as gamification, animated multimedia content, and digital mobile phone-based applications designed to teach content on parasitic infections are highly effective. Limitations such as technological and economical barriers may be overcome through the incorporation of AI for a more personalized and data-driven learning experience. Overall, we argue that the implementation of diverse edutainment strategies is an effective approach to prevent parasitic infections, and may revolutionize public health efforts with sustainable outcomes.

## Supporting information

S1 Fig Flow diagram established according to PRISMA guidelines for scoping reviews and showing methodologies of database search and selection of eligible articles. (TIF)

S1 Table Keywords used in the systematic search conducted in the present scoping review. (DOCX)

S2 Table Characteristics of the eligible studies included in the present scoping review. (DOCX)
