# Supplementary figures and images for: Breaking the cycle of parasitic diseases with edutainment: The intersection of entertainment and education
Source: PLoS Negl Trop Dis. 2025 May 28;19(5):e0013072. doi: 10.1371/journal.pntd.0013072 (PMC12119011; doi:10.1371/journal.pntd.0013072)

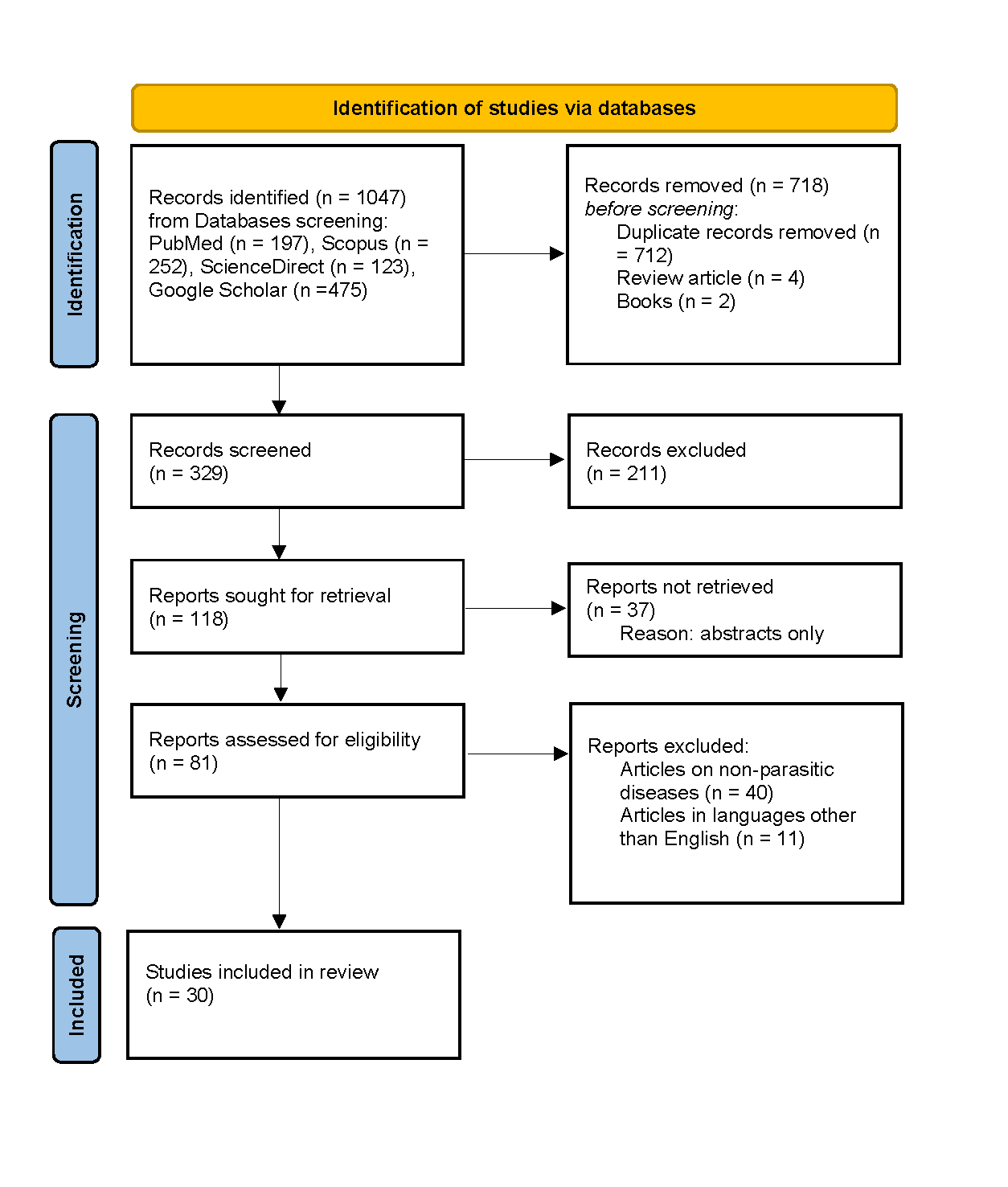

Supplement: S1 Fig — (TIF) [file pntd.0013072.s001.tif]
