# Supplementary material for: Breaking the cycle of parasitic diseases with edutainment: The intersection of entertainment and education
Source: PLoS Negl Trop Dis. 2025 May 28;19(5):e0013072. doi: 10.1371/journal.pntd.0013072 (PMC12119011; doi:10.1371/journal.pntd.0013072)
Supplement: S1 Table — (DOCX) [file pntd.0013072.s002.docx]

Supplementary Table S1. Keywords used in the systematic search conducted in the present scoping review.

| **Keyword** | **OR** | **AND** | **OR** | **AND** | **OR** |
| --- | --- | --- | --- | --- | --- |
| Edutainment | Entertainment education” | Parasites | “Parasitic Diseases” | “School children” | School |
|  | “Health education” |  | “Parasitic zoonoses” |  | Students |
|  | “Participatory methods” |  | “Soil-transmitted helminths” |  | Community |
|  | prevention |  | Ascaris |  |  |
|  | “Animated cartoon” |  | Trichuris |  |  |
|  | Games |  | Hookworms |  |  |
|  | “Computer games” |  | “Intestinal parasites” |  |  |
|  | “Board games” |  | Schistosoma |  |  |
|  | Songs |  | Schistosomiasis |  |  |
|  | Videos |  | Malaria |  |  |
|  | Music |  | “Taenia solium” |  |  |
|  | “Mobile application” |  | “Cysticercosis” |  |  |
|  | “Group discussion” |  | “Cystic echinococcosis” |  |  |
|  | Infographics |  |  |  |  |
|  | Pictures |  |  |  |  |
|  | Poster |  |  |  |  |
|  | Lectures |  |  |  |  |
|  | “PowerPoint presentation” |  |  |  |  |
|  | “Comic book” |  |  |  |  |
|  | “Educational booklet” |  |  |  |  |
|  | “Puppet show” |  |  |  |  |
|  | Toys |  |  |  |  |
|  | Drawing |  |  |  |  |
|  | Questionnaire |  |  |  |  |
|  | Quiz |  |  |  |  |
