# Supplementary material for: Breaking the cycle of parasitic diseases with edutainment: The intersection of entertainment and education
Source: PLoS Negl Trop Dis. 2025 May 28;19(5):e0013072. doi: 10.1371/journal.pntd.0013072 (PMC12119011; doi:10.1371/journal.pntd.0013072)
Supplement: S2 Table — (DOCX) [file pntd.0013072.s003.docx]

Supplementary Table S2. Characteristics of the eligible studies included in the present scoping review

| Reference | Study design  (Controlled or not) | Country | Parasite | Audience | Approach | Teacher collaboration | Preintervention testing | Postintervention testing | Treatment trials |
| --- | --- | --- | --- | --- | --- | --- | --- | --- | --- |
| Al-Delaimy et al. 2014 [1] | Yes | Malaysia | STH | School-age children | Comic book, posters, music video, puppet show, drawing activity | Yes | Questionnaire, testing children for STH | Questionnaire, testing children for STH | Yes |
| Gyorkos et al. 2013 [2] | Yes | Peru | STH | School-age children | Booklet | Yes | Questionnaire, testing children for STH |  | Yes |
| Bieri et al. 2013 [3] | No | China | STH | School-age children | Cartoon video | No | Questionnaire, drawing assessment, interviews, behavior observation | Questionnaire | No |
| Bassey et al. 2020 [4] | Yes | Nigeria | STH | School-age children | Gamification | No | Questionnaire, testing for STH | Questionnaire, testing for STH | Yes |
| Kurscheid et al. 2018 [5] | No | Indonesia | STH | Villagers 8-78 years | Puppet show | No | Questionnaire | Questionnaire | No |
| Porcu et al. 2022 [6] | No | Italy | CE | School-age children | Comic book, drawing activity, cartoon video, hands-on experience | Yes | Questionnaire | Questionnaire | No |
| Anantaphruti et al. 2008 [7] | No | Thailand | STH | School-age children | Posters, songs | Yes | Questionnaire, testing for STH | Testing for STH | Yes |
| Essé et al. 2017 [8] | No | Cote d` Ivoire | STH | School-age children | Animated cartoon | No | Questionnaire | Questionnaire | No |
| Long-Shan et al. 2000 [9] | Yes | China | STH | School-age children | Booklets, Animations | Yes | Questionnaire, testing for STH | Questionnaire, testing for STH | Yes |
| Albonico et al. 1996 [10] | No | Seychelles | STH | School-age children | Posters | Yes | Questionnaire, Testing for STH | Testing for STH | Yes |
| Rivero et al. 2017 [11] | Yes | Argentina | STH | School-age children | Cartoon video | Yes | Drawing activity, discussion | Questionnaire | No |
| Mationg et al. 2022 [12] | Yes | Philippines | STH | School-age children | Animated cartoon, Comic pamphlet, Drawing activity | No | Questionnaire, Testing for STH | Questionnaire, Testing for STH | Yes |
| Johansen et al. 2014 [13] | No | Denmark | Taenia solium cysticercosis | Professionals (medical doctors, veterinarians, meat inspectors), postgraduate students | Computer-based educational tool | NC | NC | NC | No |
| Uwibambe et al. 2014 [14] | No | Rwanda | Taenia solium cysticercosis | Health workers | Computer-based educational tool | No | Questionnaire | Questionnaire | No |
| Vaernewyck et al. 2020 [15] | No | Zambia | Taenia solium cysticercosis | Pork supply chain workers | Computer-based educational tool | No | Questionnaire, group discussion | Questionnaire | No |
| Ejike et al. 2017 [16] | Yes | Nigeria | Schistosoma | School-age children | Computer-based educational game | No | Questionnaire | Questionnaire, group discussion |  |
| Ejike et al. 2021 [17] | Yes | Nigeria | Schistosoma | School-age children | Computer-based educational game | No | Questionnaire, testing for urogenital schistosomiasis | Questionnaire | No |
| Mindu et al. 2020 [18] | No | South Africa | Schistosoma | School-age children | Poem, drama, song, pictures, an animated video | No | Questionnaire | Questionnaire | No |
| Sripa et al. 2016 [19] | No | Thailand | Opisthorchis | School-age children and the community | Brochures, cartoon books, posters, and documentary videos | Yes | Questionnaire, testing for O. viverrini | Questionnaire, testing for O. viverrini | Yes |
| Manana et al. 2021 [20] | No | South Africa | Malaria | Adults > 18 years | Songs | No | Questionnaire | Questionnaire | No |
| Guspianto et al. 2023 [21] | No | Indonesia | STH | School-age children | Computer-based educational game | NC | NC | NC | No |
| Ayi et al. 2010 [22] | Yes | Ghana | Malaria | School-age children and the community | Posters | Yes | Questionnaire | Questionnaire | No |
| Nguyen et al. 2024 [23] | No | Vietnam | Clonorchis sinensis | Adults > 18 years | Lectures, posters | NA | Questionnaire | Questionnaire | No |
| Altintas et al. 2021[24] | No | Turkey | CE | School-age children, general public, health professionals | PowerPoint presentations, posters | Yes | Questionnaire | Questionnaire | No |
| Stothard et al. 2016 [25] | No | Zanzibar | Schistosomiasis | School-age children | Comics and educational booklets | Yes | Questionnaire | Questionnaire | No |
| Howe et al. 2009 [26] | No | Hawaii | Angiostrongylus cantonensis, rat lung worm | School-age children | Puppet show | Yes | NC | NC | No |
| Yevstigneyeva et al. 2014 [27] | No | Mexico | Triatomine vectors and Chagas disease | School-age children | Drawing activities | No | NC | NC | No |
| Lorenzo et al. 2019 [28] | No | Philippines | STH, Schistosomiasis | School-age children, parents, guardians | Group discussion | No | Questionnaire | No | No |
| Ndossi et al., 2024 [29] | No | Tanzania | STH | Community | Group discussion | NA | Questionnaire, Interview, testing environmental samples | No | No |
| van Wijk et al. 2021 [30] | No | Colombia | Cutaneous leishmaniasis, Chaga`s disease | Infected persons | Group discussion | NA | Interviews | No | No |

NC, not clear.

NA, not applicable.

References

1. Al-Delaimy AK, Al-Mekhlafi HM, Lim YA, Nasr NA, Sady H, Atroosh WM, et al. Developing and evaluating health education learning package (HELP) to control soil-transmitted helminth infections among Orang Asli children in Malaysia. Parasit. Vectors.2014 Dec;7:1-8. doi. 10.1186/1756-3305-7-416
2. Gyorkos TW, Maheu-Giroux M, Blouin B, Casapia M. Impact of health education on soil-transmitted helminth infections in schoolchildren of the Peruvian Amazon: a cluster-randomized controlled trial. PLOS Negl. Trop. Dis. 2013;12;7(9):e2397. doi. 10.1371/journal.pntd.0002397
3. Bieri FA, Yuan LP, Li YS, He YK, Bedford A, Li RS, et al.. Development of an educational cartoon to prevent worm infections in Chinese schoolchildren. Infect. Dis. Poverty. 2013;2:1-9. doi. 10.1186/2049-9957-2-29
4. Bassey DB, Mogaji HO, Dedeke GA, Akeredolu-Ale BI, Abe EM, Oluwole AS, et al.. The impact of Worms and Ladders, an innovative health educational board game on Soil-Transmitted Helminthiasis control in Abeokuta, Southwest Nigeria. PLOS Negl. Trop. Dis. 202025;14(9):e0008486. doi. 10.1371/journal.pntd.0008486
5. Kurscheid J, Bendrups D, Susilo J, Williams C, Amaral S, Laksono B, et al.. Shadow puppets and neglected diseases: Evaluating a health promotion performance in rural Indonesia. Int. J. Environ. Res. Public Health. 2018;15(9):2050. doi. 10.3390/ijerph15092050
6. Porcu F, Cantacessi C, Dessì G, Sini MF, Ahmed F, Cavallo L, et al.. 'Fight the parasite': raising awareness of cystic echinococcosis in primary school children in endemic countries. Parasit. Vectors. 2022;15(1):449. doi. 10.1186/s13071-022-05575-2
7. Anantaphruti MT, Waikagul J, Maipanich W, Nuamtanong S, Watthanakulpanich D, Pubampen S, et al. School-based health education for the control of soil-transmitted helminthiases in Kanchanaburi province, Thailand. Ann. Trop. Med. Parasitol.. 2008;102(6):521-8. doi. 10.1179/136485908X311768
8. Essé C, Koffi VA, Kouamé A, Dongo K, Yapi RB, Moro HM, et al. “Koko et les lunettes magiques”: An educational entertainment tool to prevent parasitic worms and diarrheal diseases in Côte d’Ivoire. PLOS Negl. Trop. Dis. 2017;11(9):e0005839. doi. 10.1371/journal.pntd.0005839
9. Long-Shan X, Bao-Jun P, Jin-Xiang L, Li-Ping C, Sen-Hai Y, Jones J. Creating health-promoting schools in rural China: a project started from deworming. Health Promot. Int.. 2000;15(3):197-206. doi. 10.1093/heapro/15.3.197
10. Albonico M, Shamlaye N, Shamlaye C, Savioli L. Control of intestinal parasitic infections in Seychelles: a comprehensive and sustainable approach. Bull. World Health Organ.. 1996;74(6):577. PMID: 9060217
11. Rivero MR, Salas MM, Valente R, Nores MJ, De Angelo C, Arrabal J, et al. Prevention of intestinal parasites in a tri‐border area of Latin America: Children perceptions and an integral health education strategy. Zoonoses Public Health. 2017;64(8):673-83. doi. 10.1111/zph.12365
12. Mationg ML, Williams GM, Tallo VL, Olveda RM, Aung E, Alday P, et al. “The Magic Glasses Philippines”: a cluster randomised controlled trial of a health education package for the prevention of intestinal worm infections in schoolchildren. Lancet Reg Health West Pac. 2022;18. doi. 10.1016/j.lanwpc.2021.100312
13. Johansen MV, Trevisan C, Braae UC, Magnussen P, Ertel RL, Mejer H, et al.The Vicious Worm: a computer-based Taenia solium education tool. Trends Parasitol. 2014;30(8):372-4. doi. 10.1016/j.pt.2014.06.003
14. Uwibambe E, Shyaka A, Niyotwagira E, Mutoniwase J, Fèvre EM, Quinnell RJ, et al. The Vicious Worm education tool improves the knowledge of community health workers on Taenia solium cysticercosis in Rwanda. PLOS Neglected Tropical Diseases. 2024;18(4):e0012140. doi. 10.1371/journal.pntd.0012140
15. Vaernewyck V, Mwape KE, Mubanga C, Devleesschauwer B, Gabriël S, Trevisan C. Effects of ‘The Vicious Worm’educational software on Taenia solium knowledge among key pork supply chain workers in Zambia. PLOS Negl. Trop. Dis. 2020;14(10):e0008790. doi. 10.1371/journal.pntd.0008790
16. Ejike CU, Oluwole AS, Mogaji HO, Adeniran AA, Alabi OM, Ekpo UF. Development and testing of Schisto and Ladders, an innovative health educational game for control of schistosomiasis in schoolchildren. BMC Res. Notes. 2017;10:1-9. doi. 10.1186/s13104-017-2545-5
17. Ejike CU, Oluwole AS, Omitola OO, Bayegun AA, Shoneye IY, Akeredolu-Ale BI, et al. Schisto and Ladders version 2: a health educational board game to support compliance with school-based mass drug administration with praziquantel–a pilot study. Int. Health. 2021;13(3):281-90. doi. 10.1093/inthealth/ihaa057
18. Mindu T, Kabuyaya M, Chimbari MJ. Edutainment and infographics for schistosomiasis health education in Ndumo area, Kwazulu-Natal, South Africa. Cogent Med. 2020;7(1):1794272. doi. 10.1080/2331205X.2020.1794272
19. Sripa B, Tangkawattana S, Sangnikul T. The Lawa model: A sustainable, integrated opisthorchiasis control program using the EcoHealth approach in the Lawa Lake region of Thailand. Parasitol. In. 2017;66(4):346-54. doi. 10.1016/j.parint.2016.11.013
20. Manana PN, Jewett S, Zikhali J, Dlamini D, Mabaso N, Mlambo Z, et al. “Maskandi experience”: exploring the use of a cultural song for community engagement in preparation for a pilot Sterile Insect Technique release programme for malaria vector control in KwaZulu-Natal Province, South Africa 2019. Malar. J. 2021;20(1):204. doi. 10.1186/s12936-021-03736-9
21. Guspianto G, Nina EY, Mohamad I, Shabira D. Development of Health Educational Game Application “Worm Free” Based on Android. Jambi. Medical Journal “Jurnal Kedokteran dan Kesehatan. 2023;11(2):123-35. doi. 10.22437/jmj.v11i2.24993
22. Ayi I, Nonaka D, Adjovu JK, Hanafusa S, Jimba M, Bosompem KM, et al. School-based participatory health education for malaria control in Ghana: engaging children as health messengers. Malar. J. 2010;9:1-2. doi. 10.1186/1475-2875-9-98
23. Nguyen TT, Bui DT, Losson B, Dahma H, Nguyen AT, Nhu HV, et al. Effectiveness of health education in improving knowledge, attitude and practice related to foodborne zoonotic trematodes in Vietnam, with a particular focus on Clonorchis sinensis. Trop. Med. Int. Health. 2024;29(4):280-91. doi. 10.1111/tmi.13972
24. Altintas NA, Altintas NU, Yilmaz OS, Akil M, Ozturk EA, Unver A. Educational intervention for the awareness improvement and control programme design on Echinococcosis in Izmir, Turkey. Helminthologia. 2021;58(2):152-61. doi. 10.2478/helm-2021-0013
25. Stothard JR, Khamis AN, Khamis IS, Neo CH, Wei I, Rollinson D. Health education and the control of urogenital schistosomiasis: assessing the impact of the Juma na Kichocho comic-strip medical booklet in Zanzibar. J. Biosoc. Sci. 2016;48(S1):S40-55. doi. 10.1017/S0021932016000122
26. Howe K, Bach J, DeCoito M, Frias S, Hatch R, Jarvi S. Reducing rat lungworm disease in Hawai'i through a collaborative partnership with K-12 school garden and agriculture projects. fpubh. 2018;6:203. doi. 10.3389/fpubh.2018.00203
27. Yevstigneyeva V, Camara-Mejia J, Dumonteil E. Analysis of children's perception of triatomine vectors of Chagas disease through drawings: opportunities for targeted health education. PLOS Negl. Trop. Dis. 2014;8(10):e3217. doi. 10.1371/journal.pntd.0003217
28. Lorenzo PJ, Manzanilla DR, Cortel DK, Tangog E. Community perceptions of mass drug administration for soil-transmitted helminthiasis and schistosomiasis in selected schools in the Philippines. Infect. Dis. Poverty. 2019;8:1-0. doi. 10.1186/s40249-019-0595-8
29. Ndossi BA, Mjingo EE, Park H, Lee D, Bia MM, Yang H, et al. Zoonotic Parasites and Their Association With Human Activities in Northern Tanzania: An Integrated Ecosystem Approach for One Health. J. Parasitol. Res. 2024;2024(1):8872837. doi. 10.1155/2024/8872837
30. van Wijk R, van Selm L, Barbosa MC, van Brakel WH, Waltz M, Puchner KP. Psychosocial burden of neglected tropical diseases in eastern Colombia: an explorative qualitative study in persons affected by leprosy, cutaneous leishmaniasis and Chagas disease. Glob. ment. health. 2021 Jan;8:e21. doi. 10.1017/gmh.2021.18
